# Identification of Novel Pathogenic Sequence Variants of the Mismatch Repair Genes During Screening for Lynch Syndrome in a Single Centre of Eastern Hungary

**DOI:** 10.1007/s12029-020-00359-2

**Published:** 2020-01-14

**Authors:** Gergely Kóder, Judit Olasz, Janos L. Tanyi, Erin George, László Tóth, Péter Antal-Szalmás, Béla Nagy, Tamás Bubán, Csilla András, Hilda Urbancsek, Miklós Laczik, Orsolya Csuka, László Damjanovich, Miklós Tanyi

**Affiliations:** 1 Department of Surgery, Faculty of General Medicine, Medical and Health Science Centre, University of Debrecen, Móricz Zs. Krt. 22, Debrecen, 4032 Hungary; 2 Department of Pathogenetics, National Institute of Oncology, Budapest, Hungary; 3 Department of Obstetrics and Gynecology, Division of Gynecologic Oncology, Perelman Center for Advanced Medicine, University of Pennsylvania Health System, Pennsylvania, PA USA; 4 Department of Pathology, Faculty of General Medicine, University of Debrecen, Debrecen, Hungary; 5 Department of Laboratory Medicine, Faculty of General Medicine, University of Debrecen, Debrecen, Hungary; 6 Department of Internal Medicine, Faculty of General Medicine, University of Debrecen, Debrecen, Hungary; 7 Department of Oncology, Faculty of General Medicine, University of Debrecen, Debrecen, Hungary; 8 R&D Epigenetics Department of Diagenode SA, Liège, Belgium; 9 Department of Surgery, Faculty of General Medicine, University of Debrecen, Debrecen, Hungary

**Keywords:** Lynch syndrome, MMR mutation, Colorectal cancer, Screening

## Abstract

**Introduction:**

Lynch syndrome is an autosomal dominant disorder, most frequent leading to colon cancer. Identification of patients with Lynch syndrome and screening of their family members are available prevention approach that can significantly decrease mortality. Unfortunately, routine screening still does not belong to standard of care in Hungary. In this study, we performed a comprehensive screening in order to identify patients with mismatch repair (MMR) mutation between the years of 2011 and 2014. Identified mutations were compared with those already published in the international databases.

**Patients and Methods:**

Patients who underwent treatment for colorectal cancer at the Surgical Institute of the University of Debrecen were screened using the modified Amsterdam and Bethesda Criteria. Immunohistochemistry and microsatellite analyses were performed in order to identify possible mutation carrier cases. Suspicious cases underwent DNA sequencing to detect mutations in the mismatch repair genes (h*MLH1*, h*MSH2*).

**Results:**

All together 760 colorectal cancer patients were screened. A total of 28 patients were identified as possible MMR mutation carrier and underwent further genetic evaluation. Pathogenic sequence variants of the MMR gene were found in 5 patients. Hypermethylation of the promoter region of the *hMLH1* gene was identified in 2 patients. Two out of the 5 pathogenic sequence variants of the MMR gene were first identified by our group while other 2 mutations were previously published as possible founder mutations.

**Conclusion:**

Identification of families with Lynch syndrome, while challenging because of variable phenotypes at diagnosis, is feasible with available molecular biological technologies and crucial to reduce mortality caused by this syndrome.

## Introduction

### General Characteristic of Lynch Syndrome

Hereditary nonpolyposis colorectal carcinoma (Lynch syndrome), also known as Lynch syndrome, is a highly penetrant, autosomal dominant disease involving a mutation in one of the four DNA mismatch repair (MMR) genes (*MLH1*, *MSH2*, *MSH6*, or *PMS2*) or *EPCAM*. It is the most common cause of inherited colorectal cancer (CRC), accounting for approximately 3–6% of all cases [1, 2]. In addition to an increased risk of colorectal cancer, patients with Lynch syndrome have increased susceptibility to other malignancies including endometrial cancer and to a lesser extent gastric, small bowel, ovarian, upper urinary tract, biliary tract, skin, and central nervous system cancers [3].

Depending on which MMR gene is affected, lifetime risk of CRC ranges between 80 and 90%. However, there are large differences between studies evaluating the lifetime risk [4, 5]. The most common extra colic Lynch-associated tumor is endometrial cancer which is mostly endometrioid subtype with 20–60% risk depending on the MMR gene mutation. Other extra-colonic Lynch-associated tumors like ovarian cancers are serous or mucinous type, with range from 0.3–20% of lifetime risk [6, 7]. Gastric cancers are more likely intestinal type. Its estimated lifetime risk in Lynch syndrome is 5–10% [8]. The lifetime risk of small bowel cancer is between 0.4 and 12% with the appearance in the duodenum or ileum [9]. Urinary tract tumors in Lynch syndrome are transitional cell carcinomas with the localization in the ureter and renal pelvis but not in the bladder. Lifetime risk of this cancer ranges from 0.5 to 25% [10].

Most Lynch syndrome-associated tumors are adenocarcinomas. In fact, while individuals with Lynch syndrome develop adenomas at a similar rate to general population, malignant transformation requires considerably less time [11–13]. Thus, compared with sporadic cases, Lynch syndrome-associated CRC develops at an early age (median age of 45). Furthermore, they tend to present in the proximal colon (70% are right-sided tumors), synchronous and metachronous CRCs are more common [3]. Due to these characteristics of this disease above, aggressive screening is critical to reduce mortality. Both recognition of affected patients and screening of their families as well as the detection of germline mutation of the *MMR* gene is a fundamental step. While clinical criteria for Lynch syndrome such as the Bethesda and modified Amsterdam criteria exist, it can miss up to 28–68% of the affected families [14, 15]. Suboptimal sensitivity leads to the evolution of alternative screening approaches including microsatellite instability (MSI) and immunohistochemistry (IHC) analysis for MMR proteins in CRC cases. Sensitivity for this screening approach is ~ 100% with a specificity of 93% [16]. As of 2016, multiple expert guidelines recommend universal screening of all CRC patients. Patient testing is not only implicated to rule out a germline mutation in *MMR* gene, but may also impact prognosis and guide treatment for their disease. For example, MSI-high tumors have better stage-adjusted survival rates compared with microsatellite stable disease. Further, adjuvant therapy studies have demonstrated that there is no benefit of 5-fluorouracil (5-FU)-based treatment in patients with *MMR* gene mutation. In vitro studies have demonstrated that while CRC cells with a mutation of one of the *MMR* genes might not respond to 5-FU-based treatment, they may respond better to irinotecan (CPT11)-based therapies. Further prospective clinical trials are needed to evaluate the effect of different chemotherapeutic regimes [17–19].

## Patients and Methods

### Patient Selection

All patients who underwent surgical intervention for CRC between the years of 2011 and 2014 at the Surgical Institute of the University of Debrecen were screened for Lynch syndrome using a questionnaire filled out by the physician based on anamnesis. This questionnaire included a family history expanding 3 generations applying the Amsterdam and Bethesda criteria. There are several studies that evaluated the sensitivity of the Amsterdam II and revised Bethesda criteria by performing microsatellite instability or immunohistochemistry analysis or both tests as a primary screening tool in prospective unselected series of RC patients. These studies find that sensitivity of the Amsterdam II criteria for identifying individuals with HNPCC syndrome was 40% and the sensitivity of the revised Bethesda criteria was about 90%. Using the Amsterdam II and Bethesda criterial together could increase sensibility. According to the studies, if only revised Bethesda guidelines is used, 10% of the patient who carry MMR mutation could be excluded from the further molecular testing, more likely who diagnosed with CRC in the age between of 50 and 60. [3–5] In those cases where Lynch syndrome was suspected, a paraffin-embedded, formalin-fixed tumor sample was subjected to immunohistochemical staining to evaluate the presence or absence of the nuclear protein expression of MMR proteins. A peripheral blood sample was also obtained. DNA was then isolated from both tumor and peripheral blood samples to evaluate for MSI. We applied two mononucleotide markers (BAT25, BAT26), and 3 dinucleotide markers (D2S123, D5S346, D17S25) suggested by the NCI workshop [20]. In the absence of any of the MMR nuclear protein expression or in the presence of high MSI status, patient DNA was sequenced to detect *MMR* gene mutation. Furthermore, we evaluated the hypermethylation of *MLH1* gene promoter region. Large deletions in hMLH1, hMSH2, hMSH6, and the 3′-end of EPCAM gene were also tested. The presence of *BRAF* V600E point mutation was also assessed in suspicious cases.

The study was approved by the University of Debrecen’s Institutional Review Board and the Research Ethics Committee of the Medical Research Council. All patients gave written informed consent before initiation of any study procedures.

### Immunohistochemistry

Routine 5-μm-thick, formalin-fixed, paraffin-embedded tissue sections were dewaxed, rehydrated, and treated in the microwave oven in 10 mM citrate buffer (pH 6.4) for 20 min in order to restore antigenicity. Unspecific protein binding was blocked with 1% bovine serum albumin containing PBS for 30 min at 37 °C, then slides were incubated overnight with the primary antibodies (mouse monoclonal anti-MSH2, clone 25D12, Labvision Corp., Fremont, CA, USA, at 1:100 dilutions and mouse monoclonal anti-MLH1, clone G168-15, Becton–Dickinson Biosciences, USA, at 1:100 dilutions), respectively. Primary antibodies were detected by a biotin–streptavidine detection kit (LSAB, Dako, Carpinteria, CA, USA) using VIP chromogene. The slides were counterstained with methyl green. Negative controls were stained with the omission of the primary antibodies [20].

### DNA Isolation from Paraffin-Embedded Tissue Samples and Peripheral Blood of Lynch Syndrome Patients

Paraffin-embedded cancerous tissue samples of the patients were first deparaffinized with xylol, then rehydrated with ethanol. DNA was extracted by the use of High Pure PCR Template Purification Kit (Roche Diagnostics, Mannheim, Germany) or QIAamp DNA FFPE Tissue Kit (Qiagen, Hilden, Germany). In the case of peripheral blood samples, the DNA was extracted with the use of QIAamp DNA Blood Midi or Mini kit (Qiagen, Hilden, Germany), according to the manufacturer’s instructions.

### Microsatellite Analysis

The DNA of the cancerous tissues and the corresponding blood DNA were used for testing MSI. Two mononucleotide markers (BAT25 and BAT26) and 3 dinucleotide markers (D2S123, D5S346, and D17S250) were studied according to the international reference panel recommendations by the Lynch Syndrome Microsatellite Instability Test (Roche Diagnostics GmbH, Mannheim, Germany) [13, 20]. The MSI status was assessed according to the consensus of the National Cancer Institute Workshop on Microsatellite Instability for Colorectal Cancer Detection. High level instability (MSI-H) was diagnosed when at least 30% of the examined markers presented new alleles in the tumor tissue, while low level instability (MSI-L) was observed when less than 30% of the markers carried instability [20].

### Sequencing of hMSH2 and hMLH1 Genes

All exons of hMH2 and hMLH1 genes were PCR-amplified and sequenced in both directions. Sequencing reactions were performed using BigDye Cyclce Sequencing kit v.3.1 (Applied Biosystems, Foster City, CA, USA). The semi-automated fluorescence analysis was performed by the use of ABI-PRISM 3130 Genetic Analyzer (Applied Biosystems, Foster City, CA, USA) [20].

### Testing Large Genomic Deletions

Deletions of one or more exons in h*MLH1*, h*MSH2*, and h*MSH6* genes and deletions of exons 8–9 in *EPCAM* gene were tested with multiple ligation-dependent probe amplification (MLPA) method using SALSA MLPA P003-B1 and P072-C1 kits (MRC-Holland). Following intra- and inter-sample normalization, copy number alterations were assessed according to the manufacturer’s recommendations.

### MLH1 Promoter Methylation Assay

Sodium bisulfite conversion of the tumor DNA samples was carried out with EpiTect Bisulfite Kit (Qiagen) according to the manufacturer’s instructions. We performed fluorescence-based real-time PCR assay (Methylight) to assess the methylation level of the *MLH1* promoter [21]. Primer and TaqMan probe sets designed for bisulfite-converted DNA were used: one set specific for a fully methylated sequence in the *MLH1* promoter and a reference set designed for a CpG-free region of the *COL2A1* gene to normalize for input DNA. The primer and probe sequences were previously described [22, 23]. EpiTect bisulfite-converted methylated and unmethylated human control DNA (Qiagen) were used as positive and negative controls. Real-time PCRs were carried out on ABI-7900 Sequence Detection System. The reaction mixes contained 0.5 μM of each primer, 0.2-μM probe, 4.0 mM MgCl_2_, and JumpStart Taq ReadyMix (Sigma) in a final volume of 25 μl. The thermal profile was the following: 95 °C for 5 min and 45 cycles of 95 °C for 15 s and 60 °C for 1 min. The percentage of fully methylated DNA molecules (PMR) at the targeted locus was calculated by dividing the [methylated MLH1]:[COL2A1] ratio of the sample by the [methylated MLH1]:[COL2A1] ratio of the positive control and multiplied by 100. A sample was assessed as hypermethylated if the average result of three parallel reached a minimum of 4 PMR [24].

### Detection of BRAF V600E Mutation

BRAF codon 600 was examined in DNA samples extracted from formalin-fixed paraffin-embedded tumor tissue sections. Polymerase chain reactions were performed in LightCycler2.0 instrument (Roche) using LightCycler FastStart DNA Master SYBR Green I reaction mix (Roche) containing 2.5 mM MgCl_2_ and 0.5 μM primers as previously described [25]. The cycling conditions were as follows: 95 °C for 10 min and 45 cycles of 95 °C for 5 s, 60 °C for 10 s; 72 °C for 10 s. Sequencing reactions were performed with the referred primers using BigDye Terminator v3.1 Cycle Sequencing Kit (Applied Biosystems). The reaction products were run on ABI3130 Genetic Analyzer.

## Results

Screening for patients with Lynch syndrome has been an essential part of our clinical investigation since 1997. So far, screening tests have identified a number of patients with this disease also showing new sequence variants of the MMR gene that we have published recently. [20, 26, 27] Unfortunately, in spite of our efforts to screen and follow this patient population with Lynch syndrome, this has not become a routine clinical investigation nationwide in Hungary as yet.

### Patients Identified Based on Questionnaires

A total of 760 patients with colon cancer were interviewed using the Bethesda and modified Amsterdam Criteria II between 2011 and 2014. Out of them, only 60 patients with colorectal cancer (7.9%) met the criteria to be suspicious for Lynch syndrome. Seventeen patients (28.3%) from these 60 were positive and found suspicious by modified Amsterdam criteria and 43 (71.7%) were identified by Bethesda criteria. These cases are summarized in annual breakdown in Table 1.

**Table 1:** The screened patient population between 2011 and 2014 with the number of positive cases based on modified Amsterdam and Bethesda criteria in annual breakdown

Year	Questionnaires were filled	Suspect patients	Amsterdam II positive	Bethesda positive
2011	222	19	6	13
2012	213	14	5	9
2013	206	12	2	10
2014	119	15	4	11
Summary	760	60	17	43

### Results of Immunohistochemistry and Testing for Microsatellite Instability Analysis

The IHC and MSI testings were completed in 51 of the 60 identified cases. We were unable to isolate enough tumor tissue from the surgical specimen in the 9 remaining patients due to complete pathologic response, i.e., in 7 cases as a result of pre-operative chemo-radiation. The amount of available tumor sample was not sufficient for testing in the last 2 cases. IHC detected the loss of nuclear protein expression of at least one of the evaluated *MMR* gene products in 25 (49%) of the evaluated 51 cases. Using the 5 microsatellite markers to determine MSI, there were 12 (23.5%) tumors with MSI-H status and another 5 (9.8%) with MSI-L status from the evaluated 51 cases. The other 34 (66.7%) cases were microsatellite stable (MSS) as presented on Table 2.

**Table 2:** The results of the IHC, MSI, and DNA analyses in annual breakdown

Year	Number of patients	IHC (±) *_1_	Microsatellite status	Mutation found (yes/no)
2011	6	–	4 MSI-H, 2 MSI-L	Yes (2 mutation), *_2_
	6	–	6 MSS	No
	4	+	4 MSS	No DNA analysis performed
2012	4	–	2 MSI-H, 2 MSI-L	No, *_3_
	2	–	2 MSS	No
	1	+	1 MSI-H	No
	6	+	6 MSS	No DNA analysis performed
2013	2	–	2 MSI-H	Yes (2 mutation, 1 hypermethylation of *hMLH-1* gene)
	1	+	1 MSI-H	No
	8	+	8 MSS	No DNA analysis performed
2014	2	–	1 MSI-H, 1 MSI-L	Yes (1 mutation, 1 hypermethylation of *hMLH-1* gene)
	3	–	3 MSS	No
	1	+	1 MSI-H	No
	5	+	5 MSS	No DNA analysis performed
Summary	51	25+, 26-	12 MSI-H, 5 MSI-L, 34 MSS	5 mutations found

*_1_(**−**) one or more of the evaluated MMR nuclear expression was missing, (**+)** all MMR nuclear expression was present

*_2_DNA analysis was not performed in 3 patients because peripheral blood samples were not available

*_3_DNA analysis was not performed in 2 patients because peripheral blood samples were not available

Coding exons of hMLH1 and hMSH2 genes were sequenced in cases of MSI-H and/or cases of the loss of nuclear protein expression found by IHC. All together, we selected 28 patients from this time period for further DNA analysis to perform hypermethylation testing based on the results of the questionnaires, IHC and MSI testings. We found 3 cases where MSI-H status was found in spite of the normal IHC staining. In one of these 3 cases, hypermethylation of the *hMLH1* gene promoter region was identified. In other 11 patients, we found loss of at least one of the *MMR* proteins staining by IHC, but with MSS status. None of the patients in this group had a mutation in the examined *MMR* genes. All patients who showed identified sequence variants in the *MMR* genes had both MSI-H status and loss of staining of that MMR protein by IHC (Table 2). These results suggest that MSI testing has a higher specificity compared with IHC. If we had performed only MSI testing as pre-screening in this population followed by DNA sequencing as indicated by MSI-H status, we would have received the same results.

In the group selected for DNA sequencing, there were 3 patients from 2011 and 2 from 2012 who did not have peripheral blood samples available, thus sequencing was unable to be performed. We found that 2 out of these 5 patients had MSI-H status and 3 had MSI-L status. Of these 5 patients, 3 were lost for follow-up and 2 subjects died because of rapid disease progression. Due to these 5 missing samples, we changed our blood drawing schedule. Previously, we had isolated peripheral blood mononuclear cells (PBMC) for DNA sequencing only after IHC and MSI testings were completed and the results suggested a possible mutation. Since 2013, we have begun collecting blood samples in EDTA-anticoagulated tubes immediately after operation if the patient was found positive by Bethesda or modified Amsterdam II criteria, but well before any IHC or MSI data were available. Since these changes have been set, we have not missed any further blood samples for sequencing.

### Identified Sequence Variants in the MMR Genes

During the evaluation period, we identified 5 pathogenic sequence variants of the *MMR* genes and 2 cases of promoter hypermethylation of *hMLH1* from the 28 evaluated cases. So, only 0.66% of patients who answered for the questionnaires had *MMR* mutations. A mutation of *hMLH1* gene was found in 2 cases, 2 other mutations were identified in the *hMSH2* gene, and one mutation was found in *EPCAM* gene (Table 3). Interestingly, concurrent existence of promoter hypermethylation of hMLH1 and an hMSH2 pathogenic sequence variant was found in one patient (patient 6; Table 3).

**Table 3:** The locations of the mutations and the 2 h*MLH1* promoter hypermethylation

	Sex	Age at diagnosis	Amsterdam II	Bethesda	MSI	hMLH1 promoter hypermethylation	Type of gene mutation	Type of the mutation	Mutation
1	F	44	no	yes	H	no	MLH1	Probably Founder	c.143A > C; p.Q48P
2	M	40	no	yes	H	no	MLH1	New mutation	c.901insC; p.Q301Pfs5*
3	M	43	no	yes	H	no	MSH2	Probably Founder	c.2038C > T; p.R680X
4	M	32	yes	no	H	no	EPCAM	Mutation	del.ex.8–9-3-kb downstream EPCAM
5	M	43	no	yes	H	yes		No mutation hMLH1 hypermethylation	–
6	F	30	yes	no	H	yes	MSH2	New mutation with hMLH1 hypermethylation	c.2366_2367del, c.2370_2375del; p.A789Vfs6*

### No BRAF Mutation Was Found in this Study Population

It is well known that 100% of the patients with Lynch syndrome have MSI. About 15% of sporadic CRC cases also have some MSI, which can be explained by the *hMLH1* gene promoter hypermethylation. Interestingly, 50–68% of the MSI sporadic CRC cases had the V600E mutation of the *BRAF* gene, which was found to be responsible for the development of the CpG island methylator phenotype. Somatic inactivation of the *hMLH1* gene creates a Lynch syndrome phenotype without the presence of an inherited *MMR* gene mutation. Hence, evaluation of mutations in the *BRAF* gene can be a cost-effective method to separate the MSI sporadic cases from the inherited Lynch syndrome cases [28, 29]. With the application of this method, we can avoid some of the more expensive *MMR* gene mutation evaluations. There were no *BRAF* mutations identified in our study population.

### Results of the Evaluation for Promoter Hypermethylation of the hMLH1 Gene

Two of our patients had promoter hypermethylation of the *hMLH1* gene (Table 3). Interestingly, one of these patients also had an *hMSH2* mutation. In the latter case, IHC showed loss of nuclear expression of hMSH2.

### Mutations Identified in the hMLH1 Gene

A pathogenic sequence variant of *hMLH1* gene was found in 2 patients (Table 3). In patient #1, a missense mutation in codon 48, exon 2 (c.143A > C) was identified. This mutation results in an amino acid change: from glutamine to proline (p.G48P). We have previously published this mutation as it was found in our several screened families [26, 27]. It has also been reported by others as a pathogenic sequence variant of the MMR genes causing Lynch syndrome [30], and can be found in multiple databases, such as ClinVar, dbSNP, and OMIM databases (https://www.ncbi.nlm.nih.gov/clinvar/variation/89739, https://www.ncbi.nlm.nih.gov/snp/587778914). Based on these circumstances, we suspect that this is a European founder mutation.

During the DNA sequencing of the second patient, an ins > frameshift>STOP mutation of the exon 11 of *hMLH1* gene was identified. This is a one-nucleotide insertion which causes a frameshift when an extra cytosine is inserted into the 901 position. During translation, a proline will be inserted instead of glutamine at the 301 position, and behind 5 more amino acids there will be a STOP codon. Altogether, 4 new amino acids will be expressed and then the translation will be stopped (P-X-X-X-STOP). This mutation cannot be found in any of the databases and was not published yet by other groups. We believe that it is a new pathogenic sequence variants of the MMR genes, which is responsible for the development of Lynch syndrome in this patient.

### Mutations Identified in the hMSH2 Gene

MSI-H status and absence of nuclear expression of the hMSH2 and hMSH6 proteins were found in the third patient’s tumor in Table 3. DNA sequencing revealed a pathogenic nonsense mutation in exon 13 at codon 680 in the *hMSH2* gene. An arginine to STOP codon change was caused by this mutation, which results in a cytosine-thymine change at 2038 position, thus leading to premature protein chain termination. It is a well-known pathogenic sequence variants of the MMR genes causing Lynch syndrome and has been reported several times in the literature. It can also be found in dbSNP and ClinVar databases (https://www.ncbi.nlm.nih.gov/snp/63749932, https://www.ncbi.nlm.nih.gov/clinvar/variation/36572/#summary-evidence. Patient #6 had a simultaneous *hMLH1* promoter hypermethylation and *hMSH2* gene mutation on exon 14 showing a deletion → frameshift → STOP mutation. This mutation involves multiple nucleotide (c.2366_2367del, c.2370_2375del; p.A789Vfs6*), which causes a frameshift. As a consequence, the frameshift starts at the 789 position in the protein, where valine is expressed in instead of alanine. Five more codons then follow and the sixth is a STOP codon (V-X-X-X-X-STOP). Based on the ClinVar database, this region is very variable and multiple mutations have been published, most of which were associated with Lynch syndrome (https://www.ncbi.nlm.nih.gov/clinvar?term=609309[MIM]). To the best of our knowledge, this mutation has not been published so far. We believe that this is a new pathogenic sequence variant from an unstable region in the *hMSH2* gene associated with Lynch syndrome.

### Large Deletion Identified in the EPCAM Gene

The deletion of exon 9 of the *EPCAM* gene, which is associated with the hypermethylation of the hMSH2, was found in one patient. This patient tumor was MSI-H positive and IHC showed loss of nuclear expression of both the hMSH2 and hMSH6 proteins. The 8th and 9th exons of the EPCAM gene were deleted and following exon 9, the most 3′ exon this deletion continued another 3 kb after the *EPCAM* gene creating a read-through mutation. Thus, an EPCAM-MSH2 fusion transcript was created. Several EPCAM gene deletions were published and it is well known that this generates read-through mutations [31].

### Absence of Mutations in Cases Suspicious for Lynch Syndrome

All of the 5 patients who had pathogenic sequence variants of the MMR genes in this study also had MSI-H status and absence of nuclear expression of at least one of the MMR proteins on IHC. However, there was actually a total of nine patients who were MSI-H six of which lost some of their MMR protein expression. One MSI-H patient without mutation showed hMLH1 promoter hypermethylation. We did not find any pathogenic sequence variants in the *MMR* genes or hypermethylation of the promoter region of the *hMLH1* gene in the other 3 patients. In spite of the non-mutated status, there are therapeutic and prognostic relevance of the MSI-H status as we published previously [17–19]. The MSI-H status of these patients is needed to put it into consideration when decision made for the type of adjuvant chemotherapy and follow-up schedule.

## Discussion

In summary, out of the 760 patients with CRC that we formerly screened for Lynch syndrome between the years of 2011 and 2014, 5 pathogenic sequence variants of the MMR genes and 2 cases of promoter hypermethylation of the *hMLH1* gene were identified. Interestingly, a mutation of hMSH2 and hypermethylation of the hMLH1 promoter region were present simultaneously in one patient. Two of these 5 sequence variants of the MMR genes were identified for the first time by our group. Two sequence variants of the MMR genes were previously published, frequently seen, and possibly act as founder mutations. Identification of these pathogenic sequence variants of the MMR genes, whether new or well known, has important implications in our patient population to decrease the mortality caused by Lynch syndrome.

Since clinical criteria, such as the Bethesda guidelines and modified Amsterdam criteria, have low sensitivity and may reduce the number of carriers, alternative strategies were explored to identify patients and their families with Lynch syndrome. Both IHC and MSI testings demonstrate similar sensitivity as a screening test for this disease in patients with CRC [14, 32]. In fact, during the pre-screening, we found that MSI evaluation was more specific than IHC. If we did only MSI testing, we would have received the same outcome in the screening process. Other advantages of MSI analysis over IHC include the possibility of identifying a tumor that has defective MMR but retained staining on IHC (i.e., due to non-truncating missense mutation) or one that has defective MMR as a result of mutations in other genes besides *hMLH1*, *hMSH2*, or *hMSH6* and thus would be missed by IHC [33]. If we identify a particular mutation to appear multiple times in this region, it might be advised to directly analyze patients for this mutation.

Furthermore, it is important to separate the MSI-H patients without mutations as MSI-H status is associated with therapeutic and prognostic consequences [17–19]. For example, MSI-H tumors have better stage-adjusted survival rates compared with MSS disease and appears to be more prevalent in stage II disease (~ 20%) [34]. Adjuvant therapy studies with 5-FU have reported to show little to no benefit in MSI-H tumors [35]. Retrospective analyses and in vitro studies, on the other hand, have demonstrated potential benefits of other agents. Retrospective analyses suggest some potential advantage of adding oxaliplatin to 5-FU-based regimens [33] and in vitro studies suggest that these MSI-H CRC cells may respond better to irinotecan (CPT11)-based therapies [17–19]. Further prospective clinical trials are needed to evaluate the effect of different chemotherapeutic regimes. Regardless, either MSI-H status or MMR deficiency is a prognostic biomarker associated with lower risk of recurrence as well as a predictive biomarker for lack of benefit from adjuvant 5-FU-based chemotherapy [36].

Limitations of this study include that we could not perform sequencing of the *MMR* and *EPCAM* genes for all patients to confirm that there were no mutations in patients with MSS status. In particular, tumors with germline *hMSH6* mutations may not show MSI-H status due to partial redundancy of MSH6 and MSH3 proteins, which can limit MSI to mononucleotide tandem repeats. Addition of other mononucleotide markers, such as BAT-40, has re-classified some tumors with MSI-L and MSS status to MSI-H and MSI-L, respectively [33].

## Conclusion

Mutation of genes in the DNA mismatch repair pathway is implicated as the cause of Lynch syndrome. Our goal by proving the presence of germline mutation in one of the different MMR gene in Lynch syndrome patients was to provide a basis for accurate identification of the disease. Identification of families with Lynch syndrome, while challenging because of variable phenotypes at diagnosis, is feasible with available molecular biological technologies and crucial to reduce mortality caused by this syndrome. It is well known that the mortality of Lynch syndrome can be effectively decreased in proven mutation carriers and their families by performing colonoscopy every 2 years, and annual gynecologic follow-up [3, 5, 17]. In spite of this fact, the screening and identification of suspicious mutation carriers are still not performed routinely in Hungary.

We have established a screening test in Eastern Hungary some years ago and were able to identify and follow multiple patients with Lynch syndrome. All together, 760 colorectal cancer patients were screened. A total of 28 patients were identified as possible MMR mutation carrier and underwent further genetic evaluation. Pathogenic sequence variants of the MMR genes were found in 5 patients. Hypermethylation of the promoter region of the *hMLH1* gene was identified in 2 patients. Two out of the 5 pathogenic sequence variants of the MMR genes were first identified by our group while other 2 sequence variants of the MMR genes were previously published as possible founder mutations. By finding 2 new mutation during comprehensive screening in order to identify patients with mismatch repair mutation, we widen the international data of the mutation types. In discovering new pathogen mutations and showing practice of the screening in our clinic, we would like to improve the efficacy of the identification of patients with Lynch syndrome.

We believe that the continuous screening of this patient population with colorectal, ovarian, and endometrial cancer and the close preventative oncologic follow-up of the new identified cases and their family members will be applied nationwide in all eastern European countries in the near future.

## Abbreviations

Lynch syndrome: hereditary nonpolyposis colorectal cancer
CRC: colorectal cancer
MMR: mismatch repair
IHC: immunohistochemistry
MSI: microsatellite instability
MSS: microsatellite stable state
MSI-L: low-grade microsatellite instability
MSI-H: high-grade microsatellite instability
5-FU: 5 fluorouracil
